# Intrastriatal transplantation of neurotrophic factor-secreting human mesenchymal stem cells improves motor function and extends survival in R6/2 transgenic mouse model for Huntington's disease

**DOI:** 10.1371/4f7f6dc013d4e

**Published:** 2012-07-10

**Authors:** Ofer Sadan, Eldad Melamed, Daniel Offen

## Abstract

Stem cell-based treatment for Huntington's disease (HD) is an expanding field of research. Although various stem cells have been shown to be beneficial in vivo, no long standing clinical effect has been demonstrated. To address this issue, we are developing a stem cell-based therapy designed to improve the microenvironment of the diseased tissue via delivery of neurotrophic factors (NTFs). Previously, we established that bone marrow derived human mesenchymal stem cells (MSCs) can be differentiated using medium based cues into NTF-secreting cells (NTF+ cells) that express astrocytic markers. NTF+ cells were shown to alleviate neurodegeneration symptoms in several disease models in vitro and in vivo, including the model for excitotoxicity. In the present study, we explored if the timing of intrastriatal transplantation of hNTF+ cells into the R6/2 transgenic mouse model for HD influences motor function and survival. One hundred thousand cells were transplanted bilaterally into the striatum of immune-suppressed mice at 4.5, 5.5 and 6.5 weeks of age. Contrary to our expectations, early transplantation of NTF+ cells did not improve motor function or overall survival. However, late (6.5 weeks) transplantation resulted in a temporary improvement in motor function and an extension of life span relative to that observed for PBS treated mice. We conclude that late transplantation of NTF+ cells induces a beneficial effect in this transgenic model for HD. Since no transplanted NTF+ cells could be detected in vivo, we suspect that the temporary nature of the beneficial effect is due to poor survival of transplanted cells. In general, we submit that NTF+ cells should be further evaluated for the therapy of HD.

## Introduction

Stem cell-based treatment for neurodegenerative diseases, among them Huntington's disease (HD), is an expanding and dynamic field of research. To date, several sources of stem cells have shown to be beneficial using *in vivo* models for HD. For example, embryonic stem cells (ESCs) were shown to be beneficial in a toxic model for HD, in which striatal lesions are induced by quinolinic acid (QA, 1, 2). However, one of the major caveats of ESC-based therapy was also observed in this study, namely the presence of non-neural tissue within the graft.

Several research groups have investigated the efficacy of mesenchymal stem cells (MSCs) in animal models of HD, both toxic and transgenic models. MSC transplantation was shown to be beneficial in the 3-nitroproprionic toxic model for HD 3. Similarly in the QA model, Jiang et al (2011) found that introducing bone marrow derived MSCs reduced motor dysfunction and striatal degeneration, an effect that was attributed to secretion of neurotrophic factors (NTFs) 32 . In addition, Lin et al. (2011) found that MSCs derived from bone marrow not only attenuated quinolinic acid (QA) induced striatal lesions, but also improved motor function in the transgenic R6/2 mouse model for HD 5 .

A different source for MSCs is adipose tissue. Adipose-derived stem cells were shown to alleviate symptoms in the QA-toxic and R6/2 transgenic models for HD, with factor secretion suggested as a likely mechanism 6. However, HD patient-derived cells did not ameliorate disease progression in the YAC128 transgenic model in the pre symptomatic phase. Nevertheless, adipose stem cell transplantation did alter the course of disease progression when transplanted at a later, symptomatic phase, an effect that was attributed to secretion of trophic factors 7.

In another level of experiments, the clinical one, fetal striatal grafts gave rise to inconsistent results. Although some beneficial effect was suggested in several cases at first, long term follow up found reduced clinical change and increased graft degeneration 8, 9.

We have shown previously that following a medium-based differentiation process, MSCs can be induced to become neurotrophic factor-secreting cells (NTF^+^ cells). These cells not only secrete NTFs such as glial derived neurotrophic factor (GDNF) and brain derived neurotrophic factor (BDNF), but also express astrocytic markers. Additionally, we found that these differentiated cells migrate *in vivo* towards a QA-induced striatal lesion 10. Moreover, we recently demonstrated that these cells are protective *in vivo* in the QA model, as assessed by behavioral, imaging and histological parameters. Notably, HD patient-derived MSCs were found capable of differentiating efficiently into NTF^+^ cells, and protected against a QA-induced lesion similarly to cells derived from healthy people 11. This novel cell-based treatment was also shown to be effective using other models for neuronal damage, such as the 6-hydroxy dopamine model for Parkinson's disease 12, after optic nerve transaction 13, and after sciatic nerve injury 14.

In light of these promising results, especially in the HD model, we sought to discover if NTF^+^ cells are beneficial in a transgenic model for HD. In the current study we show that NTF^+^ cells represent a superior treatment as compared to untreated MCSs, however the magnitude of the beneficial effect is dependent on the timing of transplantation.

## Materials and methods

The University Committee of Animal Use for Research and Education approved all experimental protocols. The rodents were placed under 12 hour light/dark conditions and housed in individually ventilated cages with *ad libitum* access to food and water. Every effort was taken to reduce the number of animals required and to minimize their suffering.

**MESENCHYMAL STEM CELL PRODUCTION AND GROWTH**

**Mouse MSCs.** The mice were sacrificed using CO_2_ and then the tibiae and femora were dissected and placed in HBSS (Biological industries, Beit-Haemek, Israel). The epiphyses of the bones were removed and the marrow flushed out using a syringe filled with HBSS. Whole bone marrow was mechanically dissociated, washed and plated in a polystyrene plastic flask (75 cm^2^, Corning, NY) in growth medium consisting of alpha modified Eagle's medium (aMEM, Biological industries) supplemented with 20% fetal calf serum (FCS, Biological industries), 2 mM L-glutamine and SPN (both from Biological industries). The next day, the media plus any non-adherent cells were removed and the adherent cells placed in fresh medium. Cells were cultured for 2 weeks at 37^o^C in a humidified 5% CO_2_ incubator, with medium changes twice a week. Confluent cultures (passage 0) were sub-cultured according to experimental requirements. Cells used for experiments were between passages 2-6.

**Rat MSCs.** Following sacrifice of the rats with CO_2_, tibiae and femora were dissected and bones were placed in HBSS, epiphyses of the bones were removed, and the marrow was flushed out using a syringe filled with HBSS. Low-density bone marrow mononuclear cells were separated using a density gradient in UNISEP maxi tubes (NovaMed, Jerusalem, Israel). Next, the mononuclear cells (at a density of approximately 250,000/cm^2^) were plated in a polystyrene plastic flask (75 cm^2^) in growth medium consisting of DMEM (Biological industries) supplemented with 15% FCS, 2mM L-glutamine, SPN, and 0.001% 2- mercaptoethanol (Sigma-Aldrich, USA). The next day, the media plus any non-adherent cells were removed and the adherent cells placed in fresh medium. Cells were cultured for 2 weeks at 37^o^C in a humidified 5% CO_2_ incubator, with medium changes twice a week. Confluent cultures (passage 0) were sub-cultured according to experimental requirements. Cells used for experiments were between passages 2-6.

**Human MSCs.** Fresh bone marrow aspirates were harvested from iliac crests or the sternum of healthy adult donors following informed consent, under the approval of the institutional Helsinki committee of Laniado Medical Center (Natanya, Israel) or Rabin Medical Center (Petah-Tikva, Israel). Samples were diluted with HBSS. Mononuclear cells were separated using a density gradient and UNISEP maxi tubes (NovaMed, Jerusalem, Israel). Mononuclear cells were plated in polystyrene plastic 75cm^2^ tissue culture flasks in MSC growth medium, which contains DMEM, 2mM glutamine, SPN, 10% Platelet lysate (Plt), 4ul/ml Heparin, non-essential amino acids (X1) and 2-mercaptoethanol (0.001%). Platelet lysate was processed from frozen-thawed human platelet rich plasma (PRP) as described previously 12. After 24 hours non-adherent cells were removed. The medium was changed every 3-4 days. Adherent cells were cultured to 70%-90% confluence and then reseeded at a density of 5,000-10,000 cells/cm^2^. From the first passage, cell were grown in a medium containing DMEM, 2mM glutamine, SPN, heparin (4ug/ml) and 5% Plt. Cells were maintained at 37°C in a humidified 5% CO_2_ incubator. Cells used for experiments were between passages 2-6.

**INDUCTION OF NEUROTROPHIC FACTORS- SECRETING CELLS (NTF^+^CELLS)**

MSCs were placed into step one medium, which consists of DMEM supplemented with SPN, 2mM L-glutamine, 20ng/ml human epidermal growth factor (hEGF, R&D Systems, Minneapolis, MN, USA), 20ng/ml human basic fibroblast growth factor (hbFGF, R&D Systems) and 10μL/ml N2 supplement (insulin 5μg/ml, progesterone 20nM, putrescin 100μM, selenium 30nM, transferrin 100μg/ml, Invitrogen, Carlsbad, CA, USA). 72 hours later, the MSCs were placed into step two medium consisting of DMEM supplemented with SPN, 2mM L-glutamine, 1mM dibutyryl cyclic AMP (dbcAMP), 0.5mM isobutylmethylxanthine (IBMX, Both from Sigma-Aldrich), 20 ng/ml hbFGF, 50ng/ml human neuregulin1-β1 and 5 ng/ml Platelet-Derived Growth Factor-AA (PDGF, Peprotech). MSCs grown in serum-free medium containing DMEM, glutamine and SPN served as untreated controls. Due to species-specific toxicity, when inducing mouse MSCs the concentrations of IBMX and cAMP were halved in the step two medium.

**ELISA measurement of NTF secretion.** At the end of the NTF^+^ induction process, human GDNF and BDNF concentrations in the cell culture supernatant were measured using a sandwich ELISA procedure according to the manufacturer’s instructions (DuoSet, R&D System for human BDNF and GDNF). Briefly, triplicate samples were taken of the supernatant (100µl) from each flask (n=3 different donors). The samples were incubated overnight in plates coated with anti-NTF antibody before being exposed to a secondary antibody and the NTF detected by means of straptavidin-HRP (using H_2_O_2_ and tetramethylbenzidine solution). The absorbance at 450 nm and 570 nm was recorded using a Microplate Reader. The levels of secreted NTF were calculated for one million cells.

**Human stem cell transplantation and efficacy measurement in the R6/2 transgenic model for HD.** The R6/2 transgenic mouse model was used. In this strain, the first exon of the human gene encoding for huntingin is inserted with ~150 CAG repeats. The R6/2 mice were obtained from Jackson’s laboratories, and subsequently bred and genotyped according to Jackson’s Lab’s instructions. We performed three independent experiments to evaluate the efficacy of treating the animals with either hMSCs or hNTF^+^ cells. In all experiments, following chloral hydrate anesthesia, treatment cells were transplanted bilaterally using a stereotactic frame into the striatae at the following coordinates: AP +0.7mm, Lat. 2.5mm, Ven -3.5mm. 50,000 cells/μl, a total of 2μl per side, were transplanted at a rate of 1μl/min. The needle was slowly withdrawn approximately 5 minutes later. Efficacy was evaluated by assessing motor function and survival of the transgenic mice. Motor function was assessed weekly using a rotorod test (San Diego System's ROTOR-ROD™), in which the mouse is placed on a rotating rod that is gradually accelerated, and the time interval until the animal falls is measured (latency). Two hundred and forty seconds was the maximal score; after this time interval the mouse was taken off the rod. The mouse performed three trials one after the other, once weekly and the best score was taken as representative. Along with this motor function test, survival was measured on a daily basis. Immunosuppression was induced using cyclosporin A (Novartis, 15mg/Kg), beginning from a day before the cellular treatment. Cyclosporine was administered either subcutaneously (in the earliest and latest experiment, 0.1ml/animal) or in the drinking water, which was replaced twice a week (in the second experiment).

Selected animals (one from each group, randomaly selected, in the first experiment) were anesthetized, intracardially perfused with ice cold PBS followed by 4% paraformaldehyde. Brains were removed and immersed in 4% paraformaldehyde, cryoprotected in 30% sucrose, frozen in 2-methyl-butane and cryosectioned into 10μm coronal sections. Every 10th section along the striatum was stained with DAPI (Sigma-Aldrich 1:500). In these sections the transplanted cells, pre-labled with PKH-26 (Sigma-Aldrich), were searched.

**Statistics.** Generally, the results are expressed as mean ± standard error unless otherwise stated. Student’s t-test was used to compare the means of two groups. Comparisons between several groups were performed using ANOVA with Scheffe posthoc analysis. Repeated tests (weekly rotarod) were also analyzed using the repeated measurement ANOVA test. Statistical calculations were performed using SPSS v.13. Significance was defined as p<0.05.

## Results

**The amount of BDNF and GDNF secreted by NTF^+^cells is species specific.** Using ELISA we compared the amount of BDNF and GDNF secreted by mouse, rat and human MSCs at the end of the induction of NTF^+^differentiation. The resulting NTF^+^cells from each species exhibited a significant increase in BDNF secretion. However, when secretion was normalized according to the number of cells, human NTF^+^cells were found to secrete the largest amount of BDNF; 337±450, 6573±1312 and 10,126±665 pgBDNF/million cells were secreted by C57/bl mouse, Wistar rat and human NTF^+^cells, respectively. In contrast, only human NTF^+^ cells displayed increased GDNF secretion; 150±212, 35±34 and 349±46 pgGDNF/million cells were secreted by C57/bl mouse, Wistar rat and human NTF^+^cells, respectively. In light of these data, we surmise that human MSCs undergo the differentiation process most efficiently in terms of NTF secretion (Figure 1). Therefore, we chose to use human NTF^+^cells henceforth in our experiments.

**The amount of BDNF and GDNF secreted by NTF+ cells is species specific.**
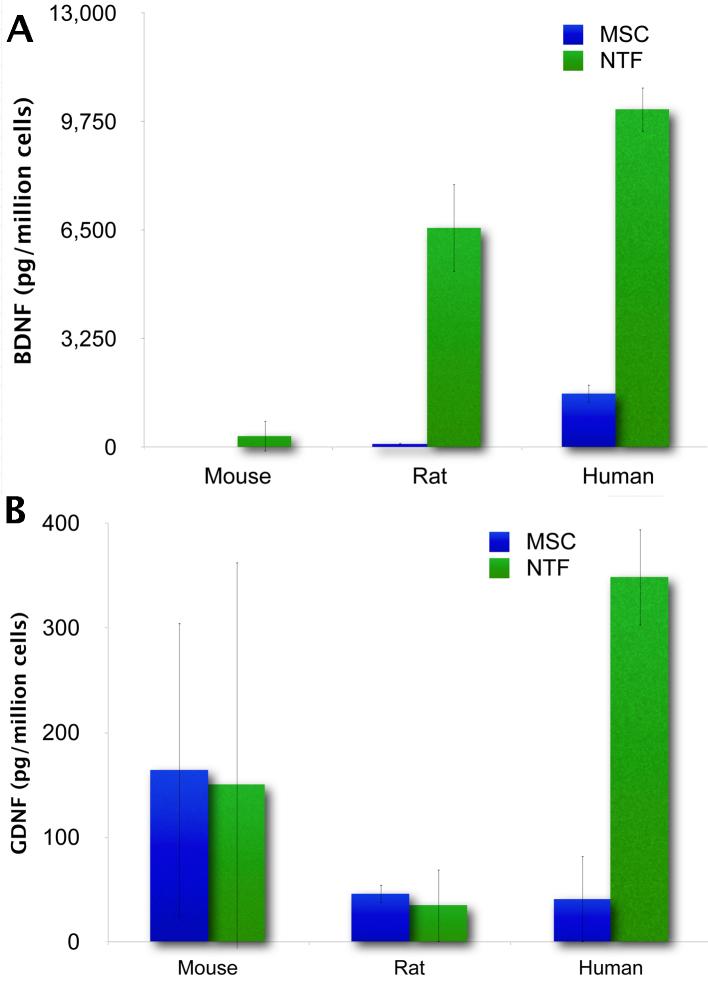
(A) BDNF secretion. (B) GDNF secretion (n=3 per species).

**Late administration of hNTF^+^cells partially ameliorates motor dysfunction in the transgenic R6/2 mouse model of HD**

Either 100,000 hMSCs (n=6) or hNTF^+^s (n=7) cells or 2µl PBS (n=7) were introduced bilaterally and intrastriatally to R6/2 mice in the 4th week of life (a week after weaning). The cellular treatment as compared to PBS had no observed effect in terms of preventing weight loss or motor deterioration (Figure 2A, rotarod assay), nor did the cellular treatment influence survival. On average, the mice survived 90.85±12.6, 88.33±18.9 and 94.43±18.1 days when treated with PBS, hMSCs or NTF^+^ cells, respectively (AVOVA p>0.05, Figure 2B). Notably, histological analysis of brain samples taken from a mouse in each group 6 weeks after treatment could not evidence surviving treatment cells (data not shown). We concluded that the treatment cells did not survive long enough to impact the htt transgene-induced neurodegeneration characteristic of R6/2 mice.

In an independent experiment, hNTF^+^ cells (n=9) or PBS (n=10) were introduced as before but in the 5^th^ week of life. No effect was detectable regarding weight (data not shown) or rotarod behavior (Figure 2C). However, the mice treated with hNTF^+^ cells lived significantly longer, surviving on average 95.4±4.0 days as compared to PBS treated mice that survived 89.5±3.2 days, (p<0.05 in a student t-test; Figure 2D).

In the next experiment, PBS (n=3), MCSs (n=4) or NTF^+^ cells (n=4) were transplanted into R6/2 mice in the 6^th^ week of life. A significant, though temporary, beneficial effect was observed in motor function (as assessed by rotorod tests) only in mice treated with NTF^+^ cells (Figure 2E). Seven days after treatment, the average latency times were 54.9±29.13 secs, 115.6±13.0 secs, 165.0±16.8 secs for PBS, MSC and NTF^+^ treated mice, respectively. Subsequently, in the 8th week of life, the average time before falling decreased considerably for PBS and MSC treated mice to 41.0±21.6s and 90.8±12.6s, respectively, but remained fairly constant for NTF^+^ cells treated mice at 143.8±14.2. In the 9th week, the PBS treated mice could remain on the rotorod only 10.2±10.2s on average before falling, whereas the latency time for the MSC and NTF^+^ cells treated mice were significantly longer at 54.6±19.2s and 92.7±13.2s, respectively. From the 10th week on, the NTF^+^ treated mice still exhibited improved motor function as compared to the PBS treated mice, but the difference in latency times was not statistically significant. In accord with the behavioral data, the survival of both MSC and NTF^+^ cells treated mice was longer relative to PBS treated mice. The MSC treated mice lived slightly longer; the average survival was 60.66, 94.5 and 91 days for the mice treated with PBS, MSCs or NTF^+^ cells, respectively (Figure 2F). Of note, a limitation of this third experiment was the small number of available animals due to inefficient breeding. This notwithstanding, taking the three experiments together, we conclude that later transplantation of stem cells is more effective. More generally, the influence of transplantation timing is likely due to issues of xenograft rejection, the temporary effect of late treatments supporting this notion.

**Cell transplantation at different time point in R6/2 transgenic mice.**
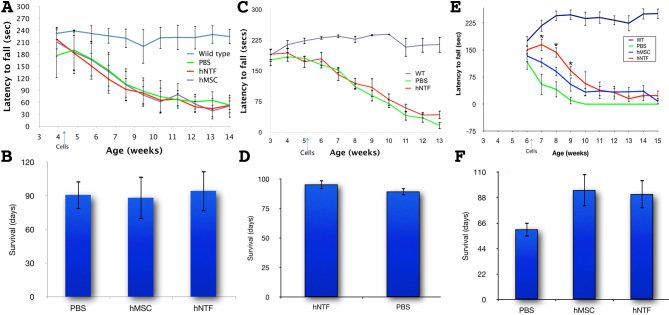
(A) Early (at 4th week) intrastriatal transplantation of hNTF+ cells does not influence motor function in the R6/2 mouse model of HD, (B) nor its survival. (C) Later (at 5th week) intrastriatal transplantation of hNTF+ cells does not alter motor function, (D) however it increases survival of R6/2 mice. (E) Late (6.5 weeks) transplantation of NTF+ cells induce a statistically significant but temporary improvement in motor function. (F) hMSC or hNTF+ cells transplantation increases life span, as compared to sham treated mice.

## Discussion

The current study evidences that human is the most promising source for NTF^+^ cells for transplantation, with respect to BDNF and GDNF secretion capacity. Moreover, this study supports that later, rather than earlier, transplantation of stem cells into the R6/2 mouse model for HD results in a more significant impact on motor function and life span.

NTFs have been linked to the pathophysiology of HD. For instance, it has been shown that BDNF transcription is influenced by the htt protein 15, 16, and in HD mice BDNF levels decrease progressively in line with disease progression 17. Indeed, a recent study highlighted the critical role of BDNF depletion in HD; the transcriptional profile of striatal tissue samples from human HD patients was shown to be more similar to that of transgenic mice depleted of BDNF than to the transcriptional profiles of known transgenic mice HD models 18.

In light of such data, NTFs are considered to represent a potential treatment for HD. Accordingly, administration of either GDNF or BDNF via a viral vector alleviates disease symptoms in the QA HD model 19 and in the 3-nitropropriyonic acid (3NP) model 20. Moreover, BDNF overexpression was reported to have a positive effect in several transgenic HD models 21, 22,23. Various approaches have been taken to indirectly increase BDNF levels and have proved efficacious in mammalian HD models 24 25 4.

The success of stem cell-based treatments in HD animal models is often attributed to secretion of NTFs 26, 27. In one effective approach, the stem cells were specifically manipulated genetically to deliver NTFs 28, 29, 30. We have taken an alternative xeno-free approach, whereby MSCs are induced to differentiate into NTF-secreting cells that express astrocytic markers, (NTF^+^ cells). This approach has several advantages. First, bone marrow derived cells have the potential to be autologous in future clinical experiments, circumventing any requirement for the immunosuppressive drugs needed for allogenic transplantation. Indeed, we have shown that HD patient-derived NTF^+^ cells are as effective as healthy donor-derived cells in behavioral, histological and imaging tests. Second, the protocol is medium based and does not involve genetic manipulation. Third, the differentiated MSCs, namely the NTF^+^ cells, exhibit almost no mitotic activity after differentiation and hence, should not be associated with any unwanted proliferation 10. Finally, in previous work, we have generated a growing body of evidence indicating that NTF^+^ cells survive after transplantation into rat brain *in vivo* and express *in vivo* at least BDNF or GDNF 11 .

In the current study, we aimed to explore the efficacy of transplanting NTF^+^ cells in a transgenic model for HD. In an unpublished experiment, we were not able to demonstrate the efficacy of syngeneic cell transplantation in the rat model for excitotoxicity, as opposed to human derived cells. Indeed, we found dramatic dissimilarities in the impact of the differentiation protocol on mouse, rat and human MSCs, though both rat and human cells increased secretion of BDNF upon differentiation. In addition, we noted that the step two medium was toxic for mouse MSCs, requiring a reduction in cAMP and IBMX concentrations. Mainly mouse, but also rat derived cells, demonstrated a markedly higher variance in the NTFs secretion levels measured, which also could be an index for the low potency of this differentiation protocol for rodent derived cells. The observed disparity in *in vitro* differentiation could be due to unknown genetic factors and/or epigenetic differences or even a difference in receptors between the species. Of note, it is conceivable that the species specific differences in secretion are an artifact of our decision to normalize NTF amounts according to the number of cells. In other words, it is possible that since the rat-derived cells are physically smaller this calculation introduces a bias in favor of human cells. However, even if this is true, since the unit of measure in the field of stem cell transplantation is a cell and not protein or RNA levels, we consider this calculation appropriate. On the basis of our calculations, we concluded that human-derived MSCs were the best candidates for studying the efficacy of NTF^+^ cell transplantation in the current study.

In the R6/2 transgenic model, the high number of CAG repeats result in clinical features that appear as early as the fourth week of life and biochemical changes that appear even earlier 31. Therefore, we expected that early administration of the cellular treatment would ameliorate behavioral changes, namely motor function, as well as elongate life expectancy. However, the empirical data support that later administration of stem cells is more effective. In light of these findings, we speculate that at early disease stages there are few chemotactic cues attracting the NTF^+^ cells to the lesion, and therefore the transplanted cells stay at the injection site and are attacked by immune cells. In contrast, at later stages, we suspect that the transplanted NTF^+^ cells migrate to diseased regions and some manage to survive. It is likely that the low survival rate of transplanted NTF^+^ cells in the mouse brain underlies the observed temporary beneficial effect. Our data accord with the findings of Im et al (2010), who reported difficulty detecting surviving transplanted cells and found that pre-symptomatic transplantation of adipose stem cells did not alter the clinical course of the YAC128 transgenic model. In contrast, late transplantation was found to induce an effect and a small number of surviving transplanted cells were detected 7.

It is noteworthy that the average survival of mice transplanted with NTF^+^ cells is longer than for PBS treated mice irrespective of whether the treatment was performed at the age of 5.5 or 6.5 weeks. Surprisingly, mice treated with PBS at 6.5 weeks of age performed more poorly in the rotorod test and exhibited a shorter life span than mice treated at earlier ages with PBS and examined at the same time point. It is difficult to ascertain the importance of these findings because of the small number of animals in each treatment group. We suspect that because the R6/2 strain is very demanding technically and has a low breeding rate, we will not be able to continue performing experiments using this particular transgenic model for HD. This notwithstanding, we are encouraged by the statistically significant results.

Future research should investigate some key unresolved issues. First, more effort should be directed towards understanding how NTF^+^ cells exert a beneficial effect and what underlies their superiority over MSCs. It remains possible that mechanisms other than NTF secretion mediate the beneficial effect, e.g., glutamate uptake, neurogenesis, induction of other cell types, such as on astrocyes, microglia etc. Second, the efficacy of our cellular technology needs to be evaluated in other transgenic models. Third, transplantation of autologous NTF^+^ cells should be explored in future clinical trials. In summary, taken together with our QA model experiments 11, the current data support further evaluation of the treatment with NTF^+^ cells for future therapy for HD.
